# QTL mapping provides new insights into emamectin benzoate resistance in salmon lice, *Lepeophtheirus salmonis*

**DOI:** 10.1186/s12864-024-11096-2

**Published:** 2024-12-18

**Authors:** Armin Sturm, Greta Carmona-Antoñanzas, Joseph L. Humble, Claudia Croton, Sally Boyd, Rapule Mphuti, John B. Taggart, David I. Bassett, Ross D. Houston, Karim Gharbi, James E. Bron, Michaël Bekaert

**Affiliations:** 1 Institute of Aquaculture, University of Stirling, Stirling, Scotland, UK; 2 The Roslin Institute and Royal (Dick) School of Veterinary Studies, University of Edinburgh, Roslin, Scotland, UK; 3 Present Address: Benchmark Holdings, Edinburgh, Scotland, UK; 4 Edinburgh Genomics, Ashworth Laboratories, King’s Buildings, University of Edinburgh, Edinburgh, Scotland, UK; 5 Present Address: Earlham Institute, Norwich, England, UK; 6 Present Address: University of Glasgow, Glasgow, Scotland, UK; 7 Present Address: Pharmaq AS, Oslo, Norway; 8 Cooke España/Culmarex, Palma, Spain

**Keywords:** Sea lice, Aquaculture, Parasite, Drug resistance, Genetics, *Lepeophtheirus salmonis*

## Abstract

**Background:**

The salmon louse (*Lepeophtheirus salmonis*) is a parasite of wild and farmed salmonid fish, causing huge economic damage to the commercial farming of Atlantic salmon (*Salmo salar*) in the northern hemisphere. The avermectin emamectin benzoate (EMB) is widely used for salmon delousing. While resistance to EMB is widespread in Atlantic populations of *L. salmonis*, the molecular mechanisms of resistance remain to be elucidated. The aim of the present work was to obtain insights into potential EMB resistance mechanisms by identifying genetic and transcriptomic markers associated with EMB resistance.

**Results:**

Crosses were performed between EMB-susceptible and -resistant *L. salmonis*, sourced from two parental strains isolated in Scotland, producing fully pedigreed families. The EMB susceptibility of individual parasites was characterised using time-to-response bioassays. Parasites of two families were subjected to double digest restriction site-associated DNA sequencing (ddRAD-seq) for simultaneous discovery of single nucleotide polymorphisms (SNPs) and genotyping. Data analysis revealed that EMB resistance is associated with one quantitative trait locus (QTL) region on *L. salmonis* chromosome 5. Marker-trait association was confirmed by genotyping assays for 7 SNPs in two additional families. Furthermore, the transcriptome of male parasites of the EMB-susceptible and -resistant *L. salmonis* parental strains was assessed. Among eighteen sequences showing higher transcript expression in EMB-resistant as compared to drug-susceptible lice, the most strongly up-regulated gene is located in the above QTL region and shows high homology to β spectrin, a cytoskeleton protein that has roles in neuron architecture and function. Further genes differentially regulated in EMB-resistant lice include a glutathione S-transferase (GST), and genes coding for proteins with predicted roles in mitochondrial function, intracellular signalling or transcription.

**Conclusions:**

Major determinants of EMB resistance in *L. salmonis* are located on Chromosome 5. Resistance can be predicted using a limited number of genetic markers. Genes transcriptionally up-regulated in EMB resistant parasites include a β spectrin, a cytoskeletal protein with still incompletely understood roles in neuron structure and function, as well as glutathione S-transferase, an enzyme with potential roles in the biochemical defence against toxicants.

**Supplementary Information:**

The online version contains supplementary material available at 10.1186/s12864-024-11096-2.

## Background

Sea lice (Caligidae, Copepoda) are ectoparasites feeding on the mucus, skin tissues and blood of marine fish [1, 2]. Caligid infections constitute a major health challenge for the mariculture of Atlantic salmon (*Salmo salar*, Linnaeus 1758), with most infections in the Northern hemisphere being caused by the salmon louse (*Lepeophtheirus salmonis*, Krøyer 1837) [3]. Depending on the severity, adverse effects of sea louse infections on the host fish include the induction of endocrine stress responses, anaemia, skin lesions, disruption of electrolyte homeostasis, loss of appetite, suppression of growth, compromised immune functions, secondary infections and, if untreated, potentially death [4]. The annual costs of sea louse infections to the salmon farming industry, comprising expenses for parasite control as well as losses in production, have been estimated at £700 M globally [5].

Salmon louse control at fish farms involves integrated pest management strategies comprising a combination of site management measures [6], veterinary treatments applied as medicinal baths or feed additives [7], and non-medicinal control approaches, such as the deployment of cleaner fish and physical delousing [8, 9]. With only a limited range of licensed anti-sea louse medicines available [3], and widespread routine use of these control agents, the risk of drug resistance formation in *L. salmonis* is significant [10].

The orally administered salmon delousing agent SLICE^®^ (MSD Animal Health) containing EMB was introduced in Norway and the UK in 1999 and 2000, respectively [3, 11]. Due to its high efficacy, including prolonged protection afforded by a one-week course of treatment, lack of side effects and convenience of in-feed administration [12, 13], EMB was extensively used during the early and mid-2000s [3, 7]. Epidemiological data suggested that by 2006, efficacy of EMB treatments had started to decline in *L. salmonis* populations affecting farmed salmon in Scotland and New Brunswick [11, 14]. In 2008 and 2009, EMB resistant *L. salmonis* strains were isolated from salmon production sites in Scotland and Norway [15, 16]. Despite EMB resistance in *L. salmonis* being currently widespread in the North Atlantic [10, 17], the molecular mechanisms underlying resistance remain to be identified.

The avermectins, which comprise ivermectin and its analogues, are widely used as anthelmintics against parasitic nematodes causing human and veterinary disease, and pesticides to control mites and insects [18, 19]. Avermectins act as allosteric modulators of glutamate-gated chloride channels (Glu-Cl) expressed in the neuromuscular systems of arthropods and nematodes, stabilising their open-pore conformation [19]. In addition, avermectins can block gamma-aminobutyric acid-gated chloride channels (GABA-Cl), which are considered secondary targets of the drug class [20]. Resistance to avermectins has evolved in several nematodes and arthropods, including nematode parasites of livestock and the phytophagous mite *Tetranychus urticae* [20, 21]. Molecular mechanisms proposed to contribute to avermectin resistance include target site mutations of Glu-Cl [22–24] as well as changes in drug metabolism such as increased expression of ATP-binding cassette (ABC) drug efflux pumps [25], cytochrome P450s [26] and/or glutathione S-transferases [27].

In *L. salmonis*, the molecular mechanisms of EMB resistance are currently poorly understood. ABC transporters [15] and ligand gated chloride channels [28] have been proposed as potential molecular determinants of EMB resistance in *L. salmonis*. However, further evidence supporting the relevance of these mechanisms for resistance in field populations of the parasite is lacking. Two microarray studies provided evidence for the up-regulation of esterase and protease transcripts in emamectin resistant *L. salmonis* [29, 30]. Another study used a single nucleotide polymorphism (SNP) array to search for genome regions showing signs of positive selection in *L. salmonis* populations of the North Atlantic [31], identifying one such region that was associated with EMB susceptibility. While EMB resistance is not widespread in *L. salmonis* populations of the Northeast Pacific Ocean, a study that applied an expanded SNP array to Pacific *L. salmonis* populations in British Columbia, Canada, found that a *L. salmonis* population at a farm site reporting EMB treatment failures was enriched for a rare genotype coinciding with the genome region associated with EMB susceptibility in Atlantic parasites [31, 32].

The aim of the present study was to obtain insights into the molecular determinants of EMB resistance in *L. salmonis*. To this end, a genotyping-by-sequencing technique [33] was applied to parasite families produced in genetic crosses between EMB susceptible and resistant parasites. The association of SNP markers with EMB resistance was assessed by quantitative trait loci (QTL) analysis. SNP markers of identified peak regions were mapped onto a chromosome-level genome assembly and the regions flanking the peak centre assessed for potential candidate genes. In addition, differential transcript expression between male *L. salmonis* of an EMB resistant strain and a drug susceptible strain was assessed using RNA-seq.

## Results

### Genetic crosses and phenotyping

**Fig. 1:**
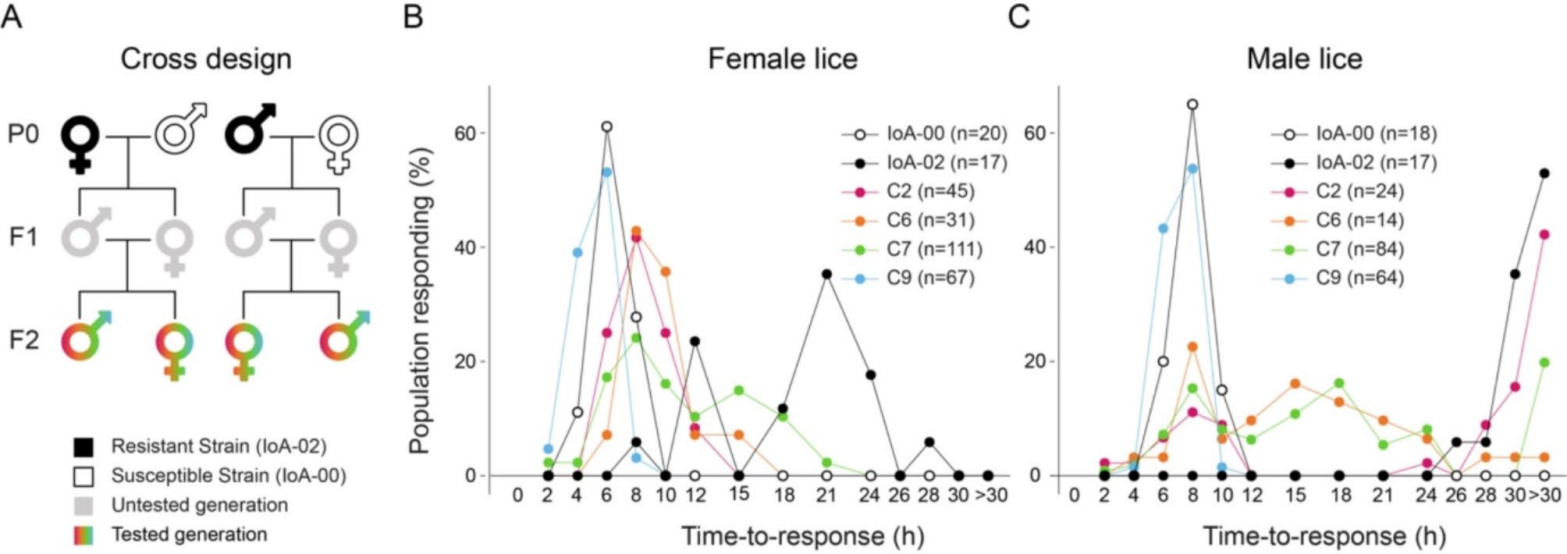
Genetic crosses and phenotyping. (**A**). Reciprocal crosses were performed between *L. salmonis* of one EMB-susceptible and one EMB-resistant strain. (**B**), (**C**). The EMB susceptibility of F2 parasites, as well as parental strain *L. salmonis*, was determined by time-to-response bioassays. Results are presented in percent of female (**B**) and male (**C**) salmon lice of the population responding

Crossing experiments (Fig. 1A) between *L. salmonis* strains IoA-00 (EMB-susceptible) and IoA-02 (EMB-resistant) produced four fully pedigreed families (C2, C6, C7, C9), each derived from a single pair of parental generation (P0) parasites. In each family, lice of the second filial generation (F2) were phenotyped using 24 h time-to-response bioassays involving exposure to 400 µg L^− 1^ of EMB (Fig. 1B and C; Supplementary Table S1). Median time-to-response (ET_50_) values were established for parental strains (IoA-00, IoA-02 lice) and F2 parasites of families generated in the crossing experiment (Table 1).

**Table 1:** Emamectin benzoate (EMB) susceptibility of *L. salmonis* strains IoA-00 and IoA-02, and F2 generation *L. Salmonis* of families derived from crosses between IoA-00 and IoA-02. EMB susceptibility was determined using time-to-response bioassay and is expressed as the median effective time (ET_50_), i.e., the time required after the beginning of waterborne exposure of animals to EMB (400 µg/L) until behavioural impairment became apparent in 50% of parasites

Strain/family	Sex	ET_50_ (h) (95% confidence)
IoA-00	Male	6.82 (6.33–7.35)
IoA-00	Female	5.21 (4.68–5.81)
IoA-02	Male	> 24 (n.d.)
IoA-02	Female	16.50 (13.91–19.56)
Family C2	Male	11.94 (9.78–14.58)
Family C2	Female	9.19 (8.50–9.94)
Family C6	Male	11.16 (9.89–12.90)
Family C6	Female	8.10 (7.17–9.15)
Family C7	Male	8.49 (4.73–15.25)
Family C7	Female	8.42 (7.59–9.35)
Family C9	Male	6.04 (5.78–6.30)
Family C9	Female	3.91 (3.59–4.25

### RAD library sequencing

High throughput sequencing of the P0, F1 and F2 parasites from families C2 and C7 (262 individuals, Supplementary Table S2) produced a total of 646,088,858 paired-end reads. After the removal of low-quality (QC < 20) and incomplete reads, 83.79% of reads were retained (541,336,509 paired-end reads). The reads were mapped against the *L. salmonis* genome assembly (GCA_905330665.1; LSAL_IOA00_R2). A total of 2,280,537 unique loci were detected, of which 129,585 were shared by at least 50% of the samples. After removing further markers in filtering and imputation steps, 84,044 common informative markers were kept and used in subsequent analyses (Supplementary Data S1).

### Association analysis and QTL mapping

**Fig. 2:**
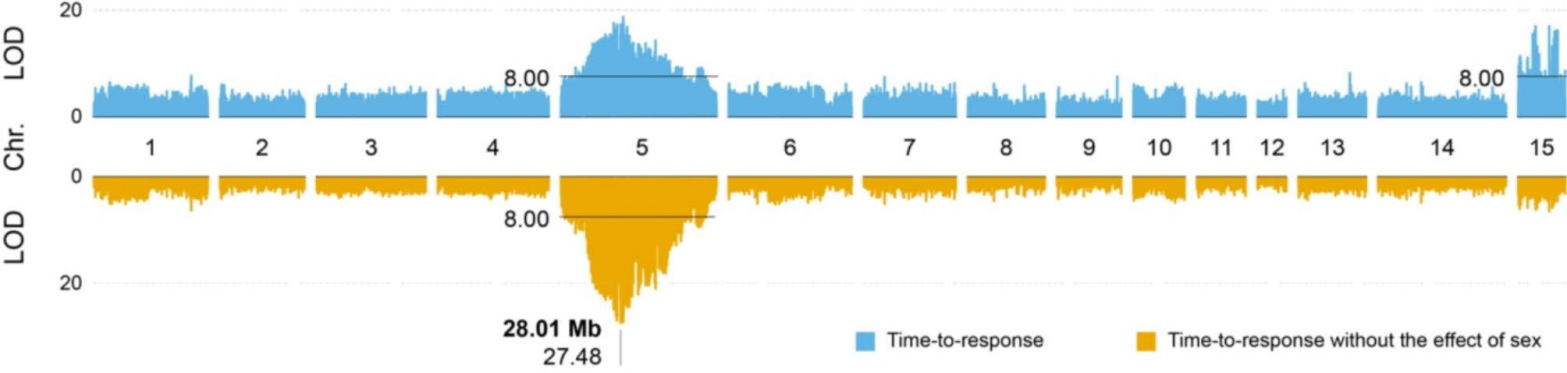
QTL mapping for the plots of LOD score along the linkage groups. Top panel: LOD score based on unfiltered time-to-response data (blue), Bottom panel: LOD score based on time-to-response data corrected for the effect of sex (orange)

**Fig. 3:**
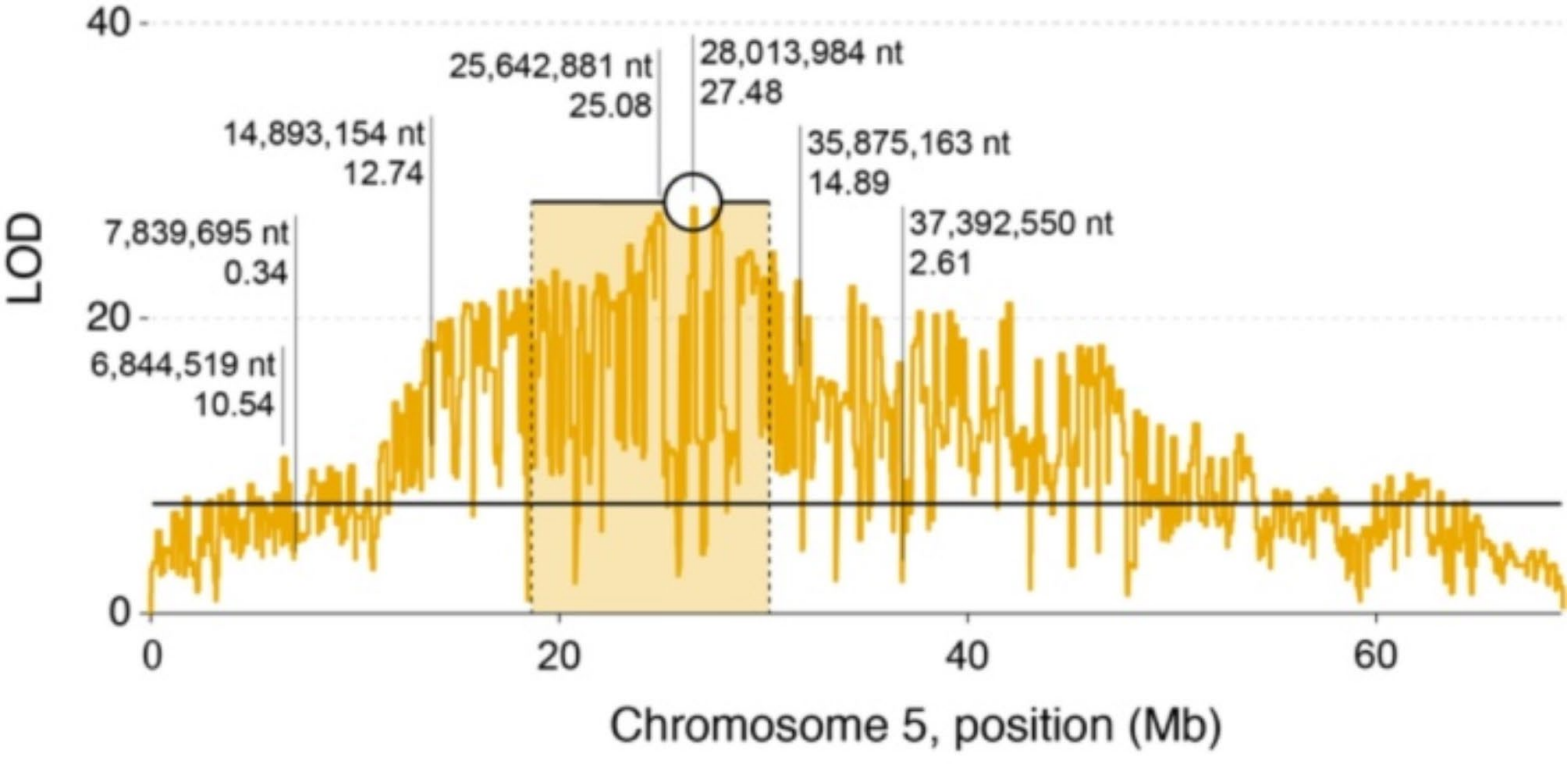
Regional analysis of the QTL for the Chromosome 5. The position of one major peak is shown as a circle, with bars indicating the respective 95% confidence intervals. Further provided are positions and LOD scores of SNP markers further investigated by allele-specific PCR assays

Using the 84,044 mapped markers, R/SNPassoc was used to conduct a quantitative trait locus (QTL) mapping analysis for the trait time-to-response. Genome-wide significant QTLs were identified on chromosomes 5 and 15 (Fig. 2, top panel). After correcting for the effect of sex on time-to-response (Fig. 2, bottom panel), only the QTL on chromosome 5 remained significant, suggesting that genetic determinants of EMB resistance are located in the region of the QTL. A more details analysis of chromosome 5 revealed one major peak (position 28,013,984, adjusted *P <* 3.33 × 10^− 28^; Fig. 3).

### Verification of SNP resistance association and susceptibility prediction

The association of selected SNP markers with EMB resistance was further investigated by allele specific genotyping assays using the KASP platform (Table 2). Markers selected for testing included 7 SNPs located on chromosome 5, of which 5 showed significant association with the EMB susceptibility. The accuracy of the KASP genotyping assays was ascertained by applying assays to at least 30 samples from families C2 and C7, for which genotypes based on ddRAD data were available (Supplementary Table S3). Identical scoring from both assay methods was obtained in all cases (data not shown). Next, *L. salmonis* of the parental strains used in the crossing experiment, as well as F2 parasites of families C6 and C9 were genotyped using KASP assays (Supplementary Table S3). For 6 of the 7 SNP loci tested, allele frequencies differed between parental *L. salmonis* strains (Supplementary Table S4). In family C6, in which individuals varied with regards to EMB susceptibility (Fig. 1B, C), 4 of the 7 SNP loci tested showed significant association with EMB susceptibility in males, while no association was found in females (Supplementary Table S5). In family C9, which was similar in bioassay responses to the EMB-susceptible parental strain IoA-00 (Fig. 1B, C), F2 animals were either homozygous at the assessed SNP loci, or no association between SNP locus genotypes and EMB susceptibility was apparent (Supplementary Table S5).

**Table 2:** SNP markers selected for allele specific PCR assays. For each marker expected association to EMB susceptibility and LOD is reported

SNP ID	Association with EMB susceptibility?	LOD	Position*	Allele A (associated phenotype)	Allele B (associated phenotype)
736518:17	Yes	10.54	6,844,519	A (EMB resistant)	G (EMB susceptible)
740175:67	No	0.34	7,839,695	G	T
765794:54	Yes	12.74	14,893,154	G (EMB resistant)	A (EMB susceptible)
802372:83	Yes	25.08	25,642,881	A (EMB resistant)	G (EMB susceptible)
810849:93	Yes	27.48	28,013,984	G (EMB resistant)	A (EMB susceptible)
839424:64	Yes	14.89	35,875,163	A (EMB resistant)	G (EMB susceptible)
844790:47	No	2.61	37,392,550	A	C

* Position on chromosome 5 (accession HG994584.1)

**Fig. 4:**
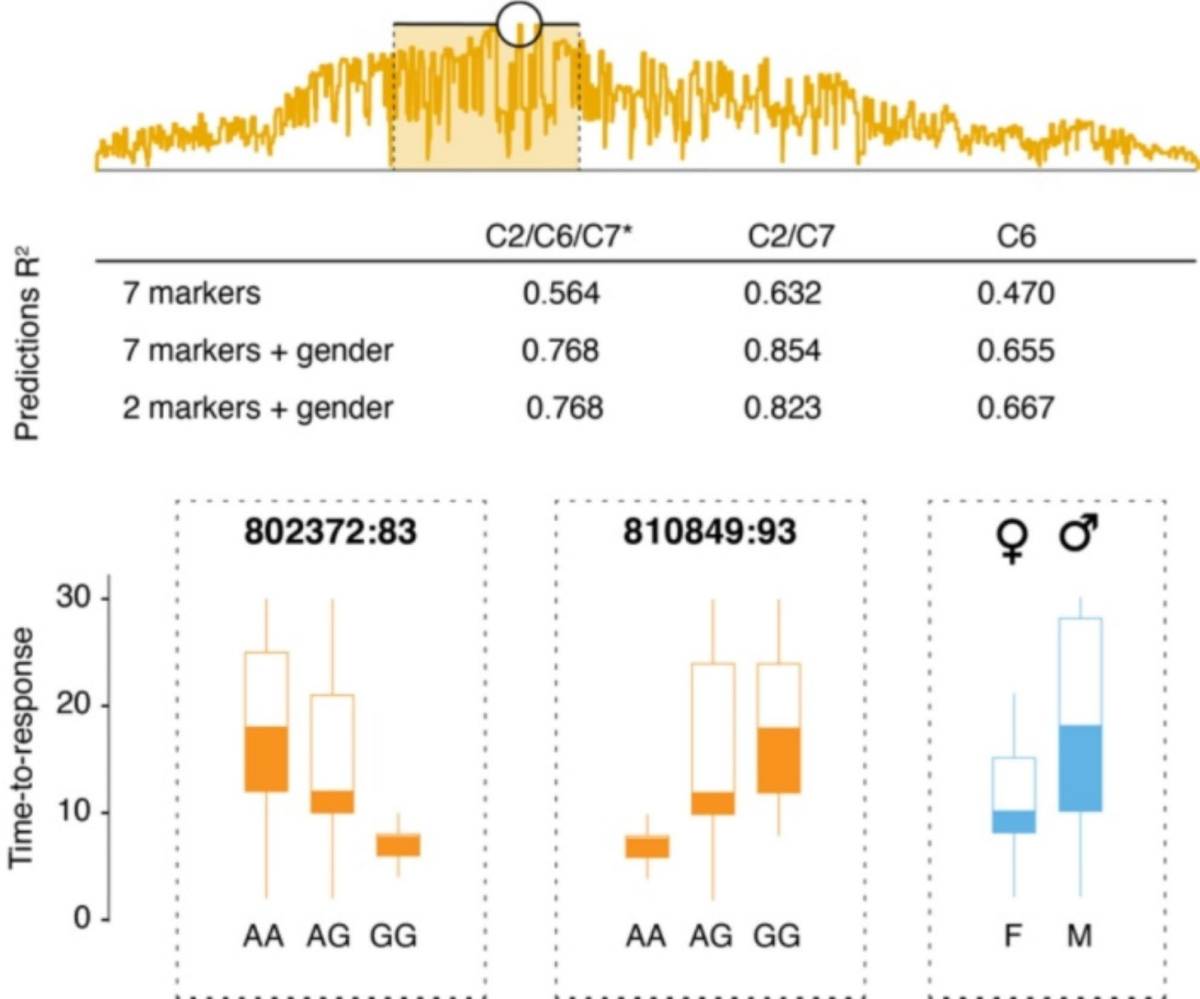
Association of SNP genotypes to EMB resistance and susceptibility prediction. Top panel: Position on Chromosome 5 of one major QTL peak and 95% confidence interval. Bottom panel: Boxplots illustrating the effect of genotype at the two most informative SNP loci on the time-to-response distribution in EMB bioassays, as well as the effect of sex, on EMB susceptibility expressed by time-to-response values. Mid panel: The predictive power of different marker combinations implemented in machine-learning algorithm, indicated by the R [2] values of correlations between predicted and observed time-to-response values. The model was trained using data from animals of families C2 and C7 (*n* = 254). Predictions were tested with either the training data set (C2/C7), or results from animals of family C6 (*n* = 44), or all data combined (C2/C7/C6). The complete set of 7 SNP markers combined achieved only moderate prediction (R^2^ = 0.564). Taking additionally into account parasite sex improved the model (R^2^ = 0.768), regardless of whether all 7 SNP markers or only the two best SNP markers were considered

Machine learning and a decision tree model were employed to predict the EMB susceptibility of individual *L. salmonis*, as apparent from their time-to-response values in bioassays, based on the animals’ genotypes at selected SNP loci. The model was trained using C2 and C7 individuals for which both time-to-response and genotype data were available (*n* = 254). Next, the performance of the model, as measured by the R [2] of the correlation between observed and predicted time-to-response values, was tested on either the training data set (C2/C7), C6 parasites (*n* = 44), or all data points (C2/C6/C7) (Fig. 4). Testing considered versions of the model using the complete set of 7 SNP markers, alone or in combination with sex, or the two best markers combined with sex. Including all 7 markers and sex, the model yielded an R [2] of 0.854 when tested on the training set and 0.655 when tested on family C6. A model based on the best two markers and sex yielded an R [2] of 0.823 with the training set and 0.667 with family C6.

### Assessment of genes in peak region

The peak region (Chromosome 5: 18,346,760–31,111,431) was examined for the presence of potential resistance genes. Most commonly, pesticide resistance in arthropods is mediated by either or both of two main molecular mechanisms, target site resistance and metabolic resistance. The effects of avermectins in ecdysozoans are believed to be mediated through interaction with the glutamate gated chloride channel (Glu-Cl), and to a lesser extent GABA-gated ion channels, of which three subtypes have been described in arthropods (RDL, Lcch3, GRD). Assessment of genes on chromosome 5 failed to identify sequences showing homology to the above types of ligand-gated ion channels. Arthropod gene superfamilies involved in metabolic pesticide resistance comprise CYPs, carboxyl esterases (CaEs), glutathione S-transferases (GSTs) and ABC (ATP binding cassette) transporters. Genes mapping to chromosome 5 did not include any CYP, CaEs or ABC sequences. Two genes showing homology to delta class GSTs (LSAL_10903, Chr 5: 49270062–49270716; LSAL_9889, Chr 5: 49284187–49287617) and one gene resembling zeta class GSTs (LSAL_9860. Chr 5: 62851048–62851999) were located on chromosome 5, but were located outside of the peak region.

### Transcriptome sequencing and assembly

Synchronised cohorts of *L. salmonis* strains IoA-00 and IoA-02 were generated to provide adult males (*n* = 8 per strain), which were subjected to transcriptome sequencing. In total, 222,097,447 and 225,569,055 raw PE-reads were obtained from strains IoA-00 and IoA-02, respectively (Table 3, Supplementary Table S6). After filtering, 201,258,349 and 204,378,124 clean PE-reads passed the mRNA cleaning step and were used for the following process. Of the clean reads, 90.62% and 90.61% were mapped to the *L. salmonis* genome assembly (GCA_905330665.1; LSAL_IOA00_R2) for IoA-00 and IoA-02 strains, respectively. A total of 21,258 transcripts were characterised and 15,356 unique genes were recovered. A BUSCO completeness assessment recovered 90.7% of near-universal single-copy orthologs selected from the metazoa database.

**Table 3:** Summary statistics of sequencing and assembly of the *L. salmonis* transcriptome generated in this study

Category	Number/length
Total number of raw PE reads	447,666,502
Maximum read length (nt)	150
Cleaned PE reads	440,909,210
Clean bases	61.2 Gb
Transcripts generated (raw)	21,258
Percentage of read assembled	90.6%
Gene (raw)	15,346
Gene (filtered)	9,169
GC content	39.6%
Maximum transcripts length	15,887
Minimum transcripts length	300
Mean length (bp)	1,670.6
Busco (Metazoa)*	887 (90.7%)

### Differential expression analysis

**Fig. 5:**
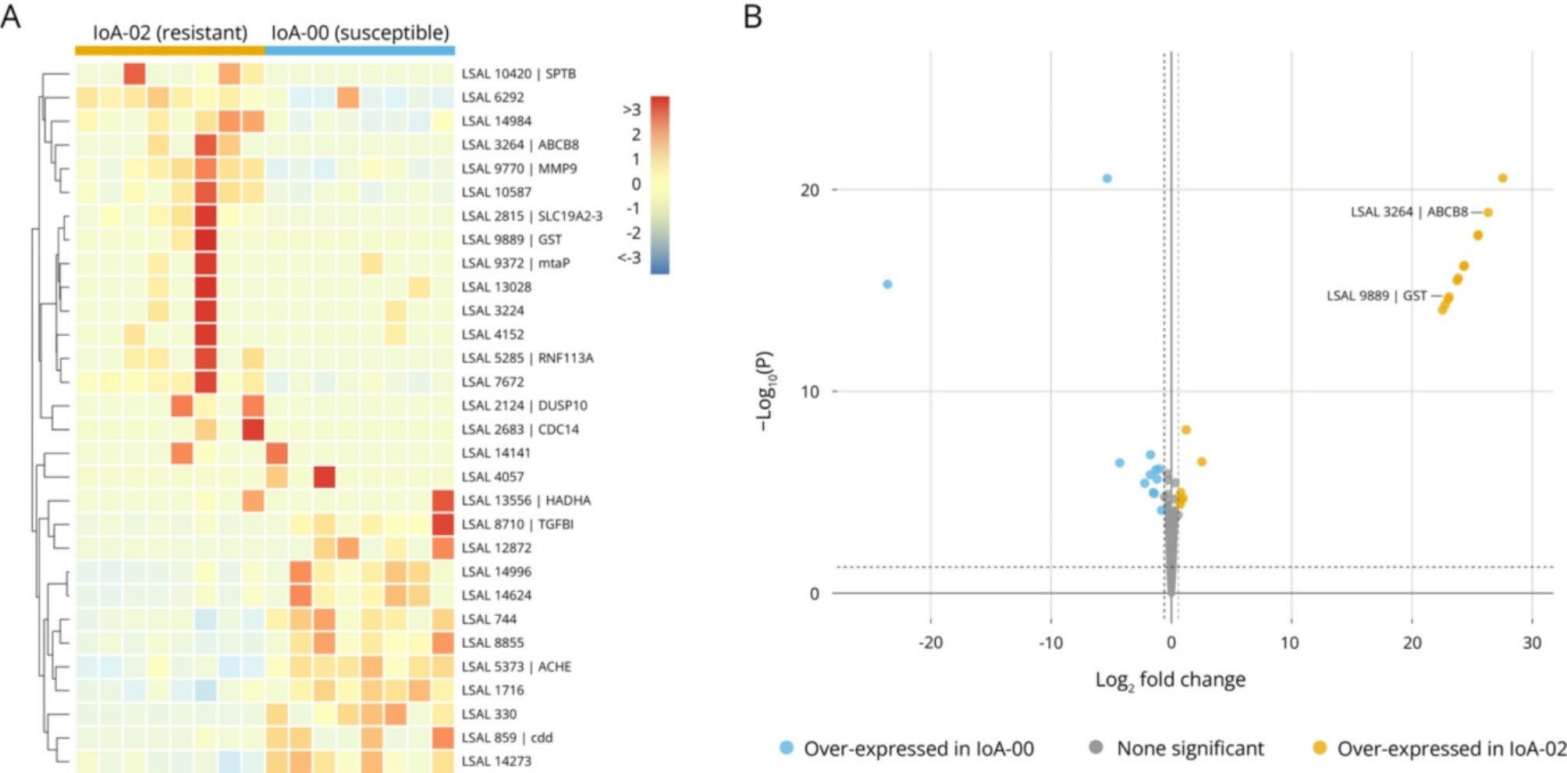
Differential gene expression. (**A**) Heatmap of the 30 genes up-/down-regulated. Gene identifiers (LSAL followed by number) are followed by their annotation, if available. SPTB: spectrin beta; ABCB8: ATP-binding cassette subfamily B (MDR/TAP) member 8; MMP9: matrix metalloproteinase-9 (gelatinase B) [EC:3.4.24.35]; SLC19A2-3: solute carrier family 19 (thiamine transporter) member 2/3; GST: glutathione S-transferase [EC:2.5.1.18]; mtaP: 5’-methylthioadenosine phosphorylase [EC:2.4.2.28]; RNF113A: RING finger protein 113 A; DUSP10: dual specificity phosphatase 10 [EC:3.1.3.16 3.1.3.48]; CDC14: cell division cycle 14 [EC:3.1.3.16 3.1.3.48]; HADHA: enoyl-CoA hydratase / long-chain 3-hydroxyacyl-CoA dehydrogenase [EC:4.2.1.17 1.1.1.211]; TGFBI: transforming growth factor-beta-induced protein; ACHE: acetylcholinesterase [EC:3.1.1.7]; cdd: cytidine deaminase [EC:3.5.4.5]. (**B**) Volcano plot of all 9,169 transcripts. Genes were selected only if *P* < 0.05 and fold-change over 1.5

A total of 15,346 genes were recovered (Supplementary Data S2), of which 9,169 showed an abundance above the threshold set for inclusion of sequences in further analysis. Differential expression analysis confirmed 30 genes to be differentially transcribed between males of strains IoA-00 and IoA-02, with 12 genes being significantly down-regulated and 18 up-regulated (Fig. 5A; Table 4, Supplementary Table S7), for which blast2GO annotation is available in Supplementary Table S8. The distribution of the changes in transcript expression is illustrated in a volcano plot (Fig. 5B). Genes showing strong transcriptional upregulation in IoA-02 (log2 fold change > 20) included sequences annotated to have functions related to cytoskeletal organisation (LSAL_10420, LSAL_2683), roles in mitochondria (LSAL_3264, LSAL_3224, LSAL_13556) or function as transcription factors (LSAL_13028, LSAOL_14984, LSAL_7672), as well as members of gene families previously associated with pesticide resistance in arthropods such as ABC transporters (LSAL_3264) and glutathione S-transferases (LSAL_9889). Both the sets of transcriptionally up- and downregulated sequences included genes predicted to be involved in protein phosphorylation or dephosphorylation (LSAL_2124, LSAL_5285, LSAL_2683, LSAL_4057), having roles in proteolysis or endopeptidase activity (LSAL_6292, LSAL_9770, LSAL_744, LSAL_14996, LSAL_14624, LSAL_12872) or acting as transporters (LSAL_2815, LSAL_10587, LSAL_1716).

**Table 4:** Genes showing differential transcript expression between male *L. salmonis* of the susceptible IoA-00 strain and the multiresistant IoA-02 strain

Gene ID	Genomic location	Fold Change (log2)	Adjusted *p*-value	Description
LSAL_10420	chr5: 20,546,176–20,548,690	2.76E + 01	1.29E-17	Spectrin beta chain-like isoform X2
LSAL_3264	chr11: 22,334,594–22,336,093	2.63E + 01	4.15E-16	Mitochondrial potassium channel ATP-binding subunit-like
LSAL_5285	chr14: 1,851,082–1,851,484	2.55E + 01	3.56E-15	Serine/threonine-protein phosphatase 1 regulatory subunit 10-like
LSAL_2124	chr1: 47,468,719–47,470,016	2.55E + 01	3.56E-15	Dual specificity protein phosphatase 10-like isoform X1
LSAL_3224	chr11: complement(13947153–13947702)	2.43E + 01	8.33E-14	Mitochondrial 28 S ribosomal protein S34
LSAL_2683	chr10: 4,051,182–4,053,470	2.43E + 01	8.33E-14	Dual specificity protein phosphatase CDC14C-like isoform X1
LSAL_4152	chr13: 1,691,001–1,691,791	2.38E + 01	2.80E-13	Keratin-associated protein 5-9-like
LSAL_13028	chr7: 15,199,095–15,199,611	2.37E + 01	3.29E-13	Transcription factor 25
LSAL_13556	chr8: 11,850,434–11,851,419	2.31E + 01	1.75E-12	Trifunctional enzyme subunit alpha, mitochondrial
LSAL_9889	chr5: 49,284,187–49,287,617	2.30E + 01	2.03E-12	Glutathione S-transferase 1-like
LSAL_9372	chr5: 34,161,984–34,162,486	2.28E + 01	3.66E-12	S-methyl-5’-thioadenosine phosphorylase
LSAL_14141	chr8 (compl.): 11,912,334–11,915,220	2.25E + 01	6.10E-12	Histone-lysine N-methyltransferase SETD7
LSAL_2815	chr11: 19,683,915–19,706,813	2.53E + 00	1.67E-04	Thiamine transporter 1-like isoform X2
LSAL_6292	chr2 (compl.): 39,538,931–39,541,895	1.23E + 00	4.92E-06	Chymotrypsin BI-like
LSAL_9770	chr5 (compl.): 42,427,793–42,439,246	9.72E-01	5.65E-03	MMP9
LSAL_14984	chr9: 1,326,958–1,336,261	8.12E-01	6.51E-03	Hypothetical predicted protein
LSAL_7672	chr3: 49,168,677–49,181,037	7.68E-01	3.37E-03	Homeobox protein DBX1-like
LSAL_10587	chr5 (compl.): 54,373,524–54,374,094	7.39E-01	9.97E-03	Facilitated trehalose transporter Tret1
LSAL_859	chr1: 9,143,842–9,144,438	-8.25E-01	1.62E-02	Cytidine deaminase-like
LSAL_14273	chr8: complement(185557–188647)	-1.01E + 00	3.36E-04	Uncharacterized transmembrane protein DDB_G0289901-like
LSAL_1716	chr1: 41,089,493–41,091,214	-1.18E + 00	8.82E-04	Solute carrier family 2, facilitated glucose transporter member 8-like
LSAL_744	chr1 (compl.):27,984,997–28,017,844	-1.29E + 00	3.52E-04	C3 and PZP-like alpha-2-macroglobulin domain-containing protein 8 isoform X2
LSAL_14996	chr9: complement(4750332–4751743	-1.42E + 00	3.68E-03	Low choriolytic enzyme-like
LSAL_14624	chr9: complement(4755875–4756900	-1.51E + 00	3.37E-03	Low choriolytic enzyme-like
LSAL_5373	chr14 (compl.): 16202224–16220752)	-1.74E + 00	7.89E-05	Acetylcholinesterase 1
LSAL_8710	chr4: 51,051,293–51,054,008	-1.76E + 00	5.71E-04	Transforming growth factor-beta-induced protein ig-h3-like isoform X1
LSAL_8855	chr4: 51,049,677–51,051,198	-2.22E + 00	1.25E-03	Embryo cathepsin L-associated protein
LSAL_12872	chr7 (compl.): 28,722,471–28,743,781	-4.30E + 00	1.78E-04	Calpain-B-like isoform X7
LSAL_330	Lsa494: 31,714–36,844	-5.33E + 00	1.29E-17	Reverse transcriptase
LSAL_4057	chr13 (compl.): 6809233–6824818)	-2.36E + 01	4.53E-13	Tissue factor pathway inhibitor isoform X2

## Discussion

The present study investigated genetic determinants of EMB resistance in *L. salmonis* derived from genetic crosses between an EMB resistant strain and a drug susceptible reference strain. After characterisation of EMB susceptibility, selected families from the cross were sequenced by ddRAD-seq to discover SNP loci and derive their genotypes. Markers associated with EMB resistance formed one QTL region on Chromosome 5. The present work confirms findings of an earlier study, which performed genome scans of SNP diversity in 12 Atlantic *L. salmonis* populations from Norwegian, Scottish, Irish, Faroese and Canadian farm sites sampled in 2009 ^31^, and found a strong selective sweep in linkage group 5 that was associated with EMB susceptibility. The QTL region identified in this study coincides with the selective sweep found previously [31], but further determined the chromosomal location of markers associated with EMB resistance.

In gastrointestinal nematodes of ruminants, points mutations of Glu-Cl [22, 34] or GABA-Cl [35] and enhanced expression of ABC transporters [25, 36] have been found to be associated with avermectin resistance. Abamectin resistance in the spider mite (*Tetranychus urticae*) has been linked to Glu-Cl mutations [23, 24] and detoxification by cytochrome P450s [26], while resistance in the silverleaf whitefly (*Bemisia tabaci*) was characterised by metabolic changes consistent with an involvement of cytochrome P450s and GSTs [27]. In the present study, chromosome 5 was assessed for the presence of different types of genes reported to be involved in avermectin resistance arthropods and nematodes. Searches failed to homologues of Glu-Cl or GABA-Cl amongst genes located on chromosome 5. Similarly, chromosome 5 did not contain any ABC transporter or CYP genes, while two sequences showing homology to GSTs were identified, both of which were outside the QTL peak region (LSAL_10903.1, chr5: 49270062–49270716; LSAL_9889.1, chr5: 49284187–49287617).

Assessing differences in transcript expression between the multiresistant strain IoA-02 and the susceptible reference strain IoA-00 using RNAseq of male parasites revealed 30 differentially regulated genes, of which 18 showed higher transcript abundances in IoA-02. While the upregulation of genes associated with pesticide resistance is commonly observed in terrestrial arthropods [37], molecular mechanisms mediating the overexpression have been studied only in a few cases and include gene duplication or gene amplification, as well as upregulation through cis- and trans-regulatory elements. The results obtained in this study do not provide evidence for mechanisms involving changes in gene copy number. Five of the differentially regulated genes map to chromosome 5, of which LSAL_10420 locates to the QTL peak region. Alterations in cis-regulatory elements located in the QTL region could have contributed to the differential expression of these genes, while trans-regulation may have contributed to the upregulation of any of the identified regulated transcripts. Taking into account that strain IoA-02 is resistant to both EMB and pyrethroids [38], some of the changes in transcript expression may be unrelated to EMB resistance and the QTL region located on chromosome 5, but instead be associated with pyrethroid resistance (discussed in more detail below).

LSAL_10420, which constitutes the most strongly upregulated transcript in strain IoA-02 and locates to the centre of the QTL region of chromosome 5, is annotated as a β spectrin (Table 4; Fig. 3). Spectrins constitute major components of the submembrane protein scaffold of animal cells also known as the membrane skeleton [39, 40]. Spectrins typically form elongated tetramers composed of α and β subunits, the length of which has been estimated to range from 50 to 200 nm [41]. In erythrocytes, spectrin tetramers interact with actin filaments to form a hexagonal mesh [39]. The architecture of the submembrane scaffold differs in axons, in which actin forms periodic submembrane rings that are interlinked by spectrin tetramers [42]. In addition to providing support to the cell membrane and allowing it to withstand mechanical challenges, spectrins of the membrane skeleton physically associate with membrane proteins, restricting their lateral mobility and contributing to the assembly of discrete plasma membrane subdomains in polarised cells including neurons. Mutations disrupting spectrin function can result in the mislocalisation of neuronal transporters and ion channels, with physiological outcomes comparable to those of transporter or channel knockouts [39, 42]. In the absence of further information about the normal role and cellular expression pattern of LSAL_10420, the physiological consequences of an overexpression of this β spectrin homologue in multiresistant *L. salmonis* and the potential mechanisms of how it may contribute to EMB resistance are difficult to judge. It is conceivable that the altered LSAL_10420 levels may lead to changes in neuronal membrane subdomains that either limit EMB interaction with its molecular targets, or mitigate the neurophysiological consequences of the drug-target interaction.

LSAL_3264, the second most up-regulated transcript in multiresistant *L. salmonis* strain IoA-02 in this study, is identical with SL-Pgp1, an ABC transporter initially suggested to represent a homologue of the drug efflux transporter MDR P-glycoprotein and to be involved in EMB resistance [43]. However, subsequent studies failed to find evidence for increased transcript expression of LSAL_3264 in EMB resistant *L. salmonis* isolates collected in Scotland [30] or New Brunswick, Canada [29]. Moreover, annotation of ABC transporters in *L. salmonis* in a previous report revealed that LSAL_3264 is a homologue of the mitochondrial protein ABCB8 [44], which is involved in the export of iron from mitochondria [45] and regulates a mitochondrial potassium channel that protects cells from oxidative stress [46, 47]. Interestingly, another transcript overexpressed in IoA-02, LSAL_3224, showed high homology to mitochondrial 298 S ribosomal protein S34. We speculate that the increased expression of transcripts LSAL_3264 and LSAL_3224, which are both annotated to have mitochondrial roles, could reflect changes in gene expression related to the strain’s pyrethroid resistance. In *L. salmonis*, deltamethrin resistance is inherited maternally and associated with mitochondrial mutations [38, 48, 49]. Based on the comparative characterisation of resistance associated mitochondrial haplotypes, a previous study concluded that a point mutation in the mitochondrial cytochrome c subunit 1 (COX1), expressed in IoA-02 and other resistant isolates, is the main genetic determinant of deltamethrin resistance in *L. salmonis* [50]. The enhanced expression of transcripts coding for mitochondrial proteins LSAL_3264 and LSAL_3224 in IoA-02 observed in this study may, therefore, reflect changes in mitochondrial gene regulation that are secondary to the above COX1 mutation.

The annotation of a number of transcripts differentially expressed between IoA-00 and IoA-02 lice would suggest their roles in cellular signalling pathways. Transcripts LSAL_2124, LSAL_5285, LSAL_2683, which show enhanced expression in drug-resistant IoA-02 lice, code for phosphatases, i.e., proteins with the role to inactivate specific target kinases. Similarly, GO terms for LSAL_4057 include protein serine/threonine kinase activity, as well as serine-type endopeptidase inhibitor activity. Transcripts LSAL_6292, LSAL_9770, LSAL_744, LSAL_14996, LSAL_14624 and LSAL_12872 are predicted to possess endopeptidase activity. The annotation of other differentially expressed transcripts suggests roles in transcription (LSAL_13028, LSAL_14984, LSAL_7672). While the above changes in transcript abundance illustrate in-depth alterations of cellular signalling potentially associated to drug resistance, specific conclusions about the pathways involved are difficult to reach in the absence of further information, such as the nature of the molecular targets of the above proteins.

Multidrug resistant *L. salmonis* studied in this report showed increased transcript expression of LSAL_9889, a GST with high homology to insect cytosolic GSTs of the delta class. Glutathione S-transferases are enzymes with roles in the detoxification of electrophilic compounds [51]. Enhanced expression of GSTs has been associated with the resistance of arthropods against organochlorines, organophosphates, pyrethroids and neonicotinoid [52, 53]. A recent report further suggested a potential involvement of arthropod GSTs in avermectin resistance [27]. GSTs can confer resistance to insecticides by different modes of action, such as conjugation to reduced glutathione, sequestration, or by mitigating oxidative stress caused by the insecticide [54].

In conclusion, this study confirms the suggestion of earlier reports that the genetic determinant(s) of EMB resistance reside on chromosome 5, and defines a QTL peak region on this chromosome. Located within the QTL peak region and showing strong overexpression in EMB resistant L. salmonis, LSAL_10420 shows high homology to β spectrins, which are cytoskeletal proteins that have been shown to be important in constituting sub-domains of the plasma membrane in neurons and are essential for the function of neuronal ion channels and transporters. More research is required to assess potential roles of β spectrin overexpression in the resistance of arthropods against avermectins. In addition, EMB resistant L. salmonis showed an increased transcript expression of LSAL_9889, a glutathione S-transferase located on chromosome 5, albeit outside the QTL peak for EMB resistance. While GSTs have been reported to contribute to the resistance of arthropods to different insecticides, potential roles of the GST LSAL_9889 in hyposensitivity of L. salmonis to EMB 4require confirmation by future studies.

## Methods

### *Lepeophtheirus salmonis* strains and husbandry

Three *L. salmonis* strains maintained in laboratory culture at the Institute of Aquaculture were used in this study. Strain IoA-00 was established in 2003 from an isolate collected in the Firth of Clyde system and is susceptible to all currently available anti-sea louse treatments including EMB [38, 55]. Strain IoA-02, which originates from the Shetland Islands and was established in 2011, is hyposensitive to EMB [55], deltamethrin [38] and azamethiphos [56]. Following isolation, strains have been maintained in the laboratory under identical conditions in the absence of drug selection, as described in detail elsewhere [15]. To maintain salmon louse cultures, gravid female lice were collected from fish anaesthetised with 2-phenoxyethanol (100 mg L^− 1^; 99%; Sigma-Aldrich, Dorset, UK). Egg-strings were removed and incubated in seawater under continuous oxygenation to allow hatching and development to infective copepodid larvae, which were then used to infect naïve salmon hosts. After successful infection, host fish were maintained under standard conditions to allow parasite development to the adult stage. Host fish carrying *L. salmonis* were inspected regularly to ensure that no spontaneous re-infections occurred, and that parasite densities remained within levels that are unlikely to significantly compromise fish welfare.

### *L. salmonis* crosses

Salmon louse crosses performed in this study comprised three generations and were run from March to October 2015. To generate families of known parentage, one pair of parental (P0) salmon lice per family was added to a tank containing one *S. salar* smolt (~ 200 g). Across the different families, drug susceptible (IoA-00) and EMB resistant (IoA-02) parents were combined in both possible sex/strain orientations. The virginity of preadult-II P0 dams used in the crosses was ascertained microscopically by confirming the absence of attached spermatophores or evidence of previous attachment. After a few days, successful mating was confirmed by visual signs of insemination in the female. At this point, the P0 sire was removed and stored in absolute ethanol for later genetic analyses. One week later, the P0 dam had produced egg strings, which were removed and incubated as described above to obtain F1 generation copepodids. The P0 dam was then sampled pending later genetic analysis while removing the genital segment to preclude contamination by male DNA from stored sperm. F1 copepodids were used to infect naïve *S. salar* smolts, on which parasites were maintained until reaching the adult male and preadult-II female stages. Using the same methodology as for P0 crosses, one breeding sibling pair of F1 parasites was set up per family in a new tank and maintained to produce F2 egg strings, which were allowed to develop to copepodids used in experimental infections designed to provide maximum numbers of F2 individuals. The F1 sire and dams, respectively, were sampled for later genetic analysis after confirmation of insemination or successful F2 copepodid production. Families were harvested and F2 lice were phenotyped by bioassay when most animals had reached the adult stage. In some families, some of the F2 parasites were at the pre-adult II stage at the time of harvest.

### Phenotyping

The EMB susceptibility of parental strain animals and F2 parasites obtained from crosses was characterised using time-to-response bioassays [55]. Prior to use in bioassays, salmon lice were collected from anaesthetised host fish as described above and allowed to recover for 2 h in seawater equilibrated to 12 °C. Emamectin benzoate (analytical grade Sigma-Aldrich, Dorset, U.K.) was solubilised using polyethylene glycol of a number average molecular weight of 300 (PEG 300, Ph Eur, Merck Chemicals, UK) before being diluted in seawater. Exposure solutions contained a final concentration of 0.05% (v/v) PEG 300. Emamectin benzoate exposures took place in 150 mL plastic Petri dishes containing 15 parasites and 70 mL of exposure solution. During the first 24 h of bioassays, animals were exposed to 400 µg L^− 1^ emamectin benzoate, followed by another 6 h of exposure to 800 µg L^− 1^ emamectin benzoate. At set time points (2, 4, 6, 8, 10, 12, 15, 18, 21, 24, 26, 28, and 30 h), the motility and attachment behaviour of salmon lice was visually examined following gentle stimulation with a fine brush. Based on the observed behaviour, parasites were assigned to categories described in detail elsewhere [55, 57] and rated “affected” or “unaffected”. Affected parasites were sampled at the first time point at which effects were noticed, taking notes of their family assignment, sex, developmental stage and time-to-response. Similarly, individuals that failed to respond until the end of the experiment were sampled with appropriate details being recorded.

### Library preparation and sequencing

Genomic DNA was extracted from individual *L. salmonis* using a salt extraction methodology, quality checked and quantified as described before [38]. Based on the pattern of EMB susceptibility distribution apparent from bioassay results, families C7 and C2 were selected for ddRAD-seq analysis. The ddRAD libraries were prepared using an adapted version of an existing protocol [58]. Briefly, DNA from each sample was digested at 37 °C for 75 min with restriction enzymes *Pst*I and *Nla*III (New England Biolabs, UK), followed by heat-inactivation at 65 °C for 25 min. The DNA samples (*n* = 262) were then individually barcoded through the ligation of specific P1 and P2 adapters each containing a unique five or seven base nucleotide sequence. After addition of pre-mixed adaptors (*Pst*I:*Nla*III 1:16) and incubation of samples at 22 °C for 10 min, T4 ligase (2000 ceU µg^− 1^ DNA), rATP (100 mM) and CutSmart buffer (1×) were added and samples were incubated for 90 min at 22 C, followed by heat inactivation (65 °C, 20 min). Samples were then pooled to produce three libraries each containing ~ 90 samples. Libraries were column purified (PCR MinElute, Qiagen), size selected by gel electrophoresis (550–650 bp) and amplified by PCR (15 cycles) [58]. Illumina sequencing (HiSeq 2500, high output run mode, v4 reagents, 125 base paired-end; three separate runs) and initial processing of raw sequence reads were performed at the Edinburgh Genomics facilities, University of Edinburgh, UK.

### RNA isolation, cDNA library construction and sequencing

Strain IoA-00 and IoA-02 animals used in RNA-seq experiments were generated in synchronised infection trials completed in March 2013. Directly after collection from host fish, parasites were removed into RNA stabilisation solution (4.54 M ammonium sulphate, 25 mM trisodium citrate, 20 mM EDTA, pH 5.4) and incubated overnight at 4 °C, before transfer to nuclease-free tubes for storage at -80 °C pending RNA extraction. The total RNA was extracted from each sample using TRIzol reagent (Invitrogen, Waltham, MA, USA) according to the manufacturer’s instructions. The purity and concentration of RNA was measured using a NanoDrop-2000 spectrophotometer (Thermo Fisher Scientific, Waltham, MA, USA) and Agilent Bioanalyzer 2100 system (Agilent Technologies, Santa Clara, CA, USA). Total RNA from eight adult male parasites of both strains were submitted to a commercial supplier (Novogene UK, Cambridge UK) for mRNA library preparation and transcriptome sequencing using the NovaSeq 6000 PE150 platform.

### Transcriptome assembly

Clean reads were obtained from the raw reads by filtering ambiguous bases, PCR duplicates, low quality sequences (QC < 20), length (150 nt), absence of primers/adaptors and complexity (entropy over 15) using fastp [59]. The remaining reads were mapped to the *L. salmonis* genome (NCBI Assembly accession GCA_905330665.1) using HiSat2 v2.2.0 [60]. The expression levels were estimated using StringTie2 v2.1.0 [61]. For each sample read coverage tables were expressed in the fragments per kilobase of exon per million mapped reads (FRKM). The longest CDS of all alternative splice-form (transcripts) sets was selected as gene. Completeness of the assembly was assessed using BUSCO v3 using the metazoa dataset [62].

### Differential expression analysis

The resulting transcript abundances for each sample were analysed using bioconductor/DESeq2 v3.10 [63]. Abundance values were normalised using variance-stabilising transformations and Binomial-Beta models. Differential expression was estimated using the function *lfcShrink* with the ashr estimator [64]; with the following thresholds to limit false positives: adjusted *P* < 0.05, fold-change > 1.5 and minimum expression > 100 FRKM for the high expressed strain. Differentially expressed transcripts were annotated using blast2GO basic v6.0.3 [65].

### Genotyping RAD alleles

The sequence data were pre-processed to discard reads of low quality (i.e., with an average quality score less than 20), lacking the restriction site or having ambiguous barcodes during samples demultiplexing stage. Retained reads were then aligned against the genomic assembly of *L. salmonis* genome (NCBI Assembly accession GCA_905330665.1) using bowtie2 v2.3.5.1 [66]. *Gstacks* from Stacks v2.62 [67] was used to identify SNPs. Bi-allelic SNPs that were common to at least 50% of the individuals using *populations* (Stacks v2.62) were filtered using PLINK v2.00a3.7LM, keeping only those with a minor allele frequency over 0.05 and not deviating from the expected Hardy-Weinberg equilibrium (adjusted *P*-value of χ [2] test ≥ 10^− 8^). Finally, Beagle v5.4-22Jul22.46e [68] was used with the GT parameter to infer missing data.

### Identification of trait associated markers

Using time-to-response determined for individual parasites in EMB bioassays as the variable indicative of EMB resistance, an association analysis was performed with the package SNPassoc v1.9-2, using the “codominant” model and R/qtl [69–71]. In the case of R/qtl the genotypes of the two families were analysed separately. The model used for the analysis was based on Interval Mapping. The algorithm considered the phenotype to follow a mixture of Bernoulli distributions and used a form of the EM algorithm for obtaining maximum likelihood estimates [72]. Two-way and multiple QTL models were also run with this package. Approximate Bayesian and 1.5-LOD 95% density and confidence intervals were calculated respectively.

### Verification of SNP association to EMB resistance

For five SNPs found to be associated with EMB resistance using the above approach, as well as two further SNPs found to lack evidence of association with this trait, marker-trait association was further tested employing allele-specific PCR assays using universal fluorescence energy transfer (FRET) probes. The primer sets for these 7 assays (Table 2) and assay components were obtained commercially (KASP v4.0, LGC Genomics). Assays were run in a 10 µL volume containing 25 ng of target gDNA and proprietary reagents following the manufacturer’s guidelines. The thermal cycling programme consisted of an initial denaturation / activation step (94℃ for 15 min), followed by 10 cycles of 20 s of denaturation (94℃) and 1 min of annealing/extension (touchdown starting at 65℃ and decreasing by 0.8℃ per cycle), and 35 cycles of 20 s denaturation and 1 min annealing / extension (57℃).

### Prediction of the EMB susceptibility phenotype

For individual salmon lice from F2 families generated in crosses, genotypes for the above 7 selected markers, obtained by ddRAD-seq or PCR assays, as well as parasite sex and (when available) time-to-response values from bioassay were entered into the WEKA package [73], which contains a variety of machine-learning algorithms, including “Decision Table” [74], an optimised rule learning algorithm. “Decision Table” then derives for each individual a predicted time-to-response value based on its genotypes for the markers considered. Permutatively, individuals were removed one-by-one from the training set, with the algorithm subsequently assigning their predicted time-to-response values.

## Electronic supplementary material

Below is the link to the electronic supplementary material.

Supplementary Material 1

Supplementary Material 2

Supplementary Material 3

Supplementary Material 4

Supplementary Material 5

Supplementary Material 6

Supplementary Material 7

Supplementary Material 8

Supplementary Material 9

Supplementary Material 10

## Data Availability

All ddRAD-seq reads generated in this study were deposited at the European Bioinformatics Institute (EBI), project PRJEB28746. RNA-seq reads were deposited at the EBI, project PRJEB41730. The datasets supporting the conclusions of this article are included within this article and its additional files.

## Abbreviations

EMB: Emamectin benzoate
MAF: Minor allele frequency
QTL: Quantitative trait locus
LG: Linkage groups
SNP: Single nucleotide polymorphisms
