# Supplementary material for: QTL mapping provides new insights into emamectin benzoate resistance in salmon lice, Lepeophtheirus salmonis
Source: BMC Genomics. 2024 Dec 18;25:1212. doi: 10.1186/s12864-024-11096-2 (PMC11657612; doi:10.1186/s12864-024-11096-2)
Supplement: Supplementary file 1 — Supplementary Material 1 [file 12864_2024_11096_MOESM1_ESM.docx]

**Table S4:** Allele frequencies of *L. salmonis* parental strains at selected SNP loci located on chromosome 5. Per strain, 24 animals (n=12 per sex) were genotyped using allele-specific PCR assays.

| SNP locus | *L. salmonis* strain | Homozygous for allele 1^1^ | Heterozygous | Homozygous for allele 2 | Frequency of allele 1 | Frequency of allele 2 | Test for difference in allele frequency between strains^2^ |
| --- | --- | --- | --- | --- | --- | --- | --- |
| 736518:17 | IOA00  IOA02 | 0  4 | 1  10 | 23  10 | 0.0208  0.3750 | 0.9792  0.6250 | P=0.00021 |
| 740175:67 | IOA00  IOA02 | 17  1 | 7  14 | 0  9 | 0.8542  0.3333 | 0.1458  0.6667 | P<0.00001 |
| 765794:54 | IOA00  IOA02 | 3  24 | 8  0 | 13  0 | 0.2917  1.0000 | 0.7083  0.0000 | P<0.00001 |
| 802372:83 | IOA00  IOA02 | 0  19 | 0  3 | 24  2 | 1.0000  0.1458 | 0.0000  0.8542 | P<0.00001 |
| 810849:93 | IOA00  IOA02 | 2  22 | 12  2 | 10  0 | 0.3333  0.9583 | 0.6667  0.0417 | P<0.00001 |
| 839424:64 | IOA00  IOA02 | 0  17 | 0  7 | 24  0 | 0.0000  0.8542 | 1.0000  0.1458 | P<0.00001 |
| 844790:47 | IOA00  IOA02 | 0  0 | 10  4 | 14  20 | 0.2083  0.0833 | 0.7917  0.9167 | P=0.15125 |

^1^ Allele 1 was associated with reduced EMB susceptibility for all SNP loci, except for loci 740175:67 and 844790:47, for which there was no association between genotype and EMB susceptibility.

^2^ Exact G test.
