# Supplementary material for: QTL mapping provides new insights into emamectin benzoate resistance in salmon lice, Lepeophtheirus salmonis
Source: BMC Genomics. 2024 Dec 18;25:1212. doi: 10.1186/s12864-024-11096-2 (PMC11657612; doi:10.1186/s12864-024-11096-2)
Supplement: Supplementary file 2 — Supplementary Material 2 [file 12864_2024_11096_MOESM2_ESM.docx]

**Table S5.** Association of SNP locus genotype and EMB susceptibility in F2 animals of families C6 and C9. For each locus, the time-to-response in EMB bioassays was compared between parasites of different genotypes (Kruskall-Wallis test, parasites of each sex tested separately).

|  | Family C6 |  |  | Family C9 |  |  |
| --- | --- | --- | --- | --- | --- | --- |
| SNP locus | Sex | N | P-value ^1^ | Sex | N | P-value |
| 736518:17 | Males | 30 | **0.048** | Males | 14 | n/a |
|  | Females | 14 | 0.391 | Females | 16 | n/a |
| 740175:67 | Males | 30 | n/a | Males | 14 | 1.000 |
|  | Females | 14 | n/a | Females | 16 | 0.703 |
| 765794:54 | Males | 30 | 0.727 | Males | 14 | 1.000 |
|  | Females | 14 | 0.823 | Females | 16 | 0.217 |
| 802372:83 | Males | 29 | **0.004** | Males | 21 | n/a |
|  | Females | 14 | 0.101 | Females | 16 | n/a |
| 810849.93 | Males | 29 | **0.007** | Males | 22 | n/a |
|  | Females | 14 | 0.21 | Females | 16 | n/a |
| 839424:64 | Males | 30 | **0.003** | Males | 22 | n/a |
|  | Females | 14 | 0.21 | Females | 16 | n/a |
| 844790:47 | Males | 30 | n/a | Males | 14 | n/a |
|  | Females | 14 | n/a | Females | 16 | n/a |

^1^n/a: Non applicable. For the given combination of locus, family and sex, all F2 individuals tested shared the same genotype, precluding comparing bioassay responses among genotypes.
